# Motivational Interviewing for Enhancing Self-care in Patients With Heart Failure: Protocol for a Randomized Controlled Trial

**DOI:** 10.2196/44629

**Published:** 2023-03-28

**Authors:** Federica Dellafiore, Greta Ghizzardi, Ercole Vellone, Arianna Magon, Gianluca Conte, Irene Baroni, Giada De Angeli, Ida Vangone, Sara Russo, Cristina Arrigoni, Rosario Caruso

**Affiliations:** 1 Department of Public Health, Experimental and Forensic Medicine Section of Hygiene University of Pavia Pavia Italy; 2 Department of Biomedicine and Prevention University of Rome Tor Vergata Rome Italy; 3 Department of Nursing and Obstetrics Wroclaw Medical University Wroclaw Poland; 4 Health Professions Research and Development Unit Istituto di Ricerca e Cura a Carattere Scientifico Policlinico San Donato San Donato Milanese Italy; 5 Department of Oncology and Hematology-Oncology European Institute of Oncology Milan Italy; 6 Nursing degree course Section Istituti Clinici di Pavia e Vigevano SPA University of Pavia Pavia Italy; 7 Department of Biomedical Sciences for Health University of Milan Milan Italy

**Keywords:** cardiology, cardiovascular, clinical trial, heart failure, motivational interviewing, randomized, heart, self-care, randomized controlled trial

## Abstract

**Background:**

Heart failure (HF) is characterized by an increasing prevalence, representing a public health problem and a significant cause of morbidity and mortality. Self-care is a cornerstone approach for optimizing therapy for patients with HF. Patients play a crucial role in managing their condition, given that several adverse health outcomes might be avoided with adequate self-care. In this regard, the literature describes motivational interviewing (MI) as highly favorable for treating chronic diseases, with promising results supporting its efficacy in enhancing self-care. Moreover, caregivers’ availability constitutes a fundamental supporting factor among the strategies to improve self-care behaviors in people with HF.

**Objective:**

The primary study aim is to test the efficacy of a structured program, including scheduled MI interventions, in improving self-care maintenance in the 3-month follow-up from the enrollment. Secondary aims comprehend the assessment of the effectiveness of the above intervention on secondary outcomes (eg, self-care monitoring, quality of life, sleep disturbance) and the corroboration of the superiority of caregivers’ participation to the intervention over the program administrated only to individual patients in enhancing self-care behaviors and other outcomes at 3, 6, 9, and 12 months from the enrollment.

**Methods:**

This study protocol designed a prospective, parallel-arm, open-label, 3-arm, controlled trial. The MI intervention will be administered by nurses trained in HF self-care and MI; the education program will be provided to nurses by an expert psychologist. Analyses will be performed within the framework of intention-to-treat analysis. Comparisons between groups will be based on an alpha of 5% and 2-tailed null hypotheses. In the case of missingness, analyzing the extent of the missingness and identifying underlying mechanisms and patterns will guide imputation methods.

**Results:**

The data collection was started in May 2017. We completed the data collection with the last follow-up in May 2021. We plan to perform data analysis by December 2022. We plan to publish the study results within March 2023.

**Conclusions:**

MI enhances potential self-care practices in patients with HF and their caregivers. Although MI is effectively largely employed either alone or combined with other treatments and is administered in different settings and ways, face-to-face interventions seem to be more effective. Dyads with higher shared HF knowledge are more efficient in promoting self-care adherence behaviors. Moreover, patients and caregivers may perceive proximity with health care professionals, resulting in a better ability to follow the received health professionals’ directions. The scheduled in-person meetings with patients and caregivers will be exploited to administer MI, respecting all the safety regulations for infection containment. The conduction of this study may support changes in clinical practice to include MI to improve self-care for patients with HF.

**Trial Registration:**

ClinicalTrials.gov NCT05595655; https://clinicaltrials.gov/ct2/show/NCT05595655

**International Registered Report Identifier (IRRID):**

DERR1-10.2196/44629

## Introduction

Heart failure (HF) is defined as “a clinical syndrome with symptoms and or signs caused by a structural and/or functional cardiac abnormality and corroborated by elevated natriuretic peptide levels and or objective evidence of pulmonary or systemic congestion” [1]. HF is characterized by an increasing prevalence and burden, representing a public health problem and a major cause of morbidity and mortality [1,2]. Globally, the overall estimated incidence of HF disease is 1-4 cases per 1000 person-years, with a prevalence of 10-30 cases per 1000 persons [3]. The incidence of the disease in Europe is about 5 per 1000 person-years, with a prevalence of 1%-2% in adults [4]. The prevalence of HF increases significantly with age: 6.6% of men with HF are aged 60 to 79 years, and 10.6% are aged 80 years and older [5].

Due to the impact of the disease, some authors have defined HF to be the “global outbreak” of the 21st century about 25 years ago [6,7]. An estimated 64.3 million people worldwide are affected by HF [7]; the growing and aging of the population, the high survival rate after an acute coronary event, and the effectiveness of secondary prevention and drug treatment result in a constant increase in the number of patients living with HF [4,7-9]. HF is most frequently diagnosed in patients who are hospitalized and 65 years and older [10]. People surviving at 1 and 5 years of follow-up have increased by about 20% compared to 1950-1970s [11]; however, 5-year mortality is still high, resulting in 24.4% for people aged 60 years and 54.4% for people aged 80 years [5].

Outcomes related to HF have improved in recent years [4]. Thus far, hospitalization and rehospitalization still impact the health care system; treating patients with HF costs US $30 billion every year, and this figure is destined to grow further, reaching US $70 billion by 2030 in the United States [5]. Once HF is first diagnosed, patients are readmitted to the hospital about once a year; this trend contributes to increasing health care resource consumption and worsens patients’ quality of life (QoL) [7,12]. Moreover, HF is associated with considerable morbidity and mortality [13]. A 60- to 90-day mortality from diagnosis is about 10% in patients with HF; 1-year mortality is about 36% [14].

Self-care is a cornerstone approach for optimizing therapy for patients with HF [15]. Self-care is defined as an active, cognitive process through which people engage to maintain their health (self-care maintenance) and recognize and manage signs and symptoms (self-care monitoring and management, respectively) [16]. Precisely, self-care maintenance is defined by behaviors to preserve physical and psychological status, self-care monitoring encompasses behaviors about the ability to recognize signs and symptoms, and self-care management consists of the ability to manage signs and symptoms when they occur [17,18]. The consequences of inappropriate self-care behaviors can be dramatic, resulting in emergency hospital admissions, recurrent hospitalizations, and preventable complications. Poor adherence to treatments and improper disease management are the main causes of the exacerbation of HF [19-21]. In this regard, patients play a key role in the management of their condition, given that several negative health outcomes might be avoided with effective self-care [20,22].

Caregivers’ availability constitutes a key supporting factor among the strategies to improve self-care behaviors in people with HF [23]. Studies involving patients with HF and their caregivers [24-26] report that self-care in HF is a “dyadic phenomenon” [27]: informal caregivers, such as a family member or a close friend, are fundamental in contributing to HF patients’ self-care, as they provide tangible and emotional support and cooperation [23]. Caregivers tend to adapt their conduct based on patients’ ability to care for themselves: they may act by reminding self-care behaviors or replacing the patient in the self-care process.

Many international studies have tested interventions to improve self-care in patients with HF involving caregivers to enhance the effectiveness of educational interventions [25,28,29]. Among the interventions reported in the literature, motivational interviewing (MI) has been described as highly favorable for treating chronic diseases, with promising results supporting its efficacy in enhancing self-care [30]. This intervention is a directive client-centered counseling approach for eliciting behavioral change by helping people to explore and resolve ambivalence. MI considers several critical factors in improving self-care in patients with HF and is based on brief interventions, which are feasible in several health care settings [30,31].

A recent study involving patients with HF and their caregivers tested the efficacy of MI in improving self-care maintenance, self-care management, and self-care confidence in adults with HF at the 3-month assessment after enrollment [32]. In this study, a 1-year follow-up was also performed, and the MI was performed only once. The influence exerted by caregivers on self-care behaviors employed by patients was also assessed using a 3-arm design [32]. More precisely, in the first arm, MI was administered to patients only; in arm 2, MI was administered to patients and caregivers; and in arm 3, participants received usual care (standard education). The study demonstrated that MI performed by trained nurses effectively improved self-care in patients with HF, especially in the arm where it was administered to both patients and caregivers [32]. However, the 1-year follow-up showed that the effects of MI on self-care confidence and on proxy measurement of specific self-care behaviors, such as physical activity and functioning, did not last over 3 months if administered only one time [32]. Similar results emerged from a recent meta-analysis [33].

Thus far, it is still unclear if administrating face-to-face MI more than once over 1 year might stabilize the positive effects of MI on self-care behaviors in patients with HF, and additional evidence is required to determine whether performing a MI on the dyad (patients and caregivers) is better than applying MI only to the individual patient [33]. For these reasons, this study protocol designed a prospective, parallel-arm, open-label, 3-arm, controlled trial for purposing the following aims: (1) to test the efficacy of a structured program including scheduled MI interventions in improving self-care behaviors (specifically, self-care maintenance) among patients with HF at 3 months from the enrollment; (2) to test the effect of a structured program including scheduled MI interventions on secondary outcomes (eg, self-care monitoring, QoL, sleep disturbance) at 3, 6, 9, and 12 months from the enrollment; and (3) to corroborate the superiority of the caregivers’ participation to the structured program including scheduled MI interventions over the program administrated only to individual patients in enhancing self-care behaviors.

## Methods

### Trial Design

This is a prospective, parallel-arm, open-label, 3-arm, controlled, and superiority randomized controlled trial (RCT). This protocol was designed and reported following the SPIRIT (Standard Protocol Items: Recommendations for Interventional Trials) 2013 statement: defining standard protocol items for clinical trials (see Multimedia Appendix 1).

### Sample and Setting

Outpatients referred to the heart failure clinic and inpatients at discharge after hospitalization for HF will be enrolled in the study. The single center where the study will be implemented is the IRCCS Policlinico San Donato, northern Italy. The recruitment strategy will be based on enrolling patients continuously until the predetermined sample size is achieved.

### Randomization, Allocation Concealment, and Blinding

Patients and caregivers will be randomized into 3 study arms by a computer software program that generates the random sequence. A simple randomization algorithm will be used to generate the sequences, and the randomization list will be concealed by using cloud-based software with restricted access only to the statistician who developed the randomization list and who will not be involved in the data analysis.

In the control group, patients receive MI, and caregivers receive traditional educational treatment. In the first interventional arm, MI will be used to reinforce the education of patients and caregivers will receive standard education. In the second interventional arm, MI will be used to reinforce self-care education in patients and caregivers. The standard educational approach will be adopted for all patients and caregivers and will be based on the preidentified informational materials about self-care in patients with HF.

The nature of the intervention does not allow researchers to employ the blindness of participants and investigators because the interventionist and the enrolled subjects will be aware of the allocated arm; in this regard, the study is considered open-label. However, blinding will be employed in the outcome assessment as the outcome assessors will have to follow predefined standard operative procedures that encompass their blinding in assessing self-care. Furthermore, blinding will be ensured during the data entry process and analytics. Data will be managed with the Research Electronic Data Capture (REDCap) platform, a secure, web-based software application and workflow methodology designed to collect and manage data for research studies.

### Inclusion and Exclusion Criteria

The following inclusion criteria will be used for patients: being 18 years and older, diagnosis of HF with New York Heart Association (NYHA) Class II-IV, no acute coronary events within 3 months, consent to participate in the study, score ≤ 2 to at least 2 items of the Self-Care of Heart Failure Index 6.2 (SCHFI 6.2) at baseline [34], scores on Six Item Screener >4 [35], living at home, understanding of spoken and written Italian. The exclusion criteria for patients will be severe cognitive impairment (score 0-4 on the Six-item Screener) [35], acute coronary events that occurred within 3 months, living in nursing homes or residential settings, and caregivers unwilling to participate in the study.

The following inclusion criteria will be used for caregivers: being 18 years or older, being the primary informal caregiver identified by the patient, consent to participate in the study, and understanding of spoken and written Italian. Their unavailability will give the exclusion criterion for caregivers to participate in the study.

### Instruments and Outcomes

The measurements for patients will be based on collecting sociodemographic and clinical characteristics data (eg, age, gender, NYHA class), SCHFI 6.2 [34], Heart Failure Somatic Perception Scale to assess patient’s ability to feel the symptoms of HF [36], Six Item Screener to identify subjects with cognitive impairment [35], Kansas City Cardiomyopathy Questionnaire (KCCQ) to assess HF-specific QoL [37], Mutuality Scale (MS) to assess the feeling of intimacy and positive relationship between patient and caregiver [38], 12-Item Short Form Survey (SF-12) to measure generic health-related QoL [39], Pittsburgh Sleep Quality Index (PSQI) to assess sleep quality [40], Hospital Anxiety and Depression Scale (HADS) to detect depression and anxiety [41], Montreal Cognitive Assessment (MoCA) to detect mild cognitive impairment [42], and Charlson Comorbidity Index (CCI) to assess comorbidities [43].

The measurements for caregivers will be based on collecting sociodemographic data (eg, age, gender, marital status, and relationship with the patient), the Caregiver Contribution to Self-care of Heart Failure Index (CC-SCHFI) to measure the caregiver contribution to self-care in patients with HF [44], SF-12 [39], MS [38], Caregiver Preparedness Scale to assess how the caregiver feels prepared to care [45], HADS [41], Multidimensional Scale of Perceived Social Support to assess the level of social support perceived by patients with HF in managing the disease [46], and PSQI [40].

All the tools used to assess both patients’ and caregivers’ outcomes are constituted by validated and widely used instruments. The SCHFI 6.2 [34] is an instrument adopted to assess patients’ self-care, which is the primary outcome. It is a widely used scale that comprises 3 subdimensions: self-care maintenance, self-care management, and self-care confidence. A 0 to 100 score can be attributed to each dimension; scores higher than 70 indicate an adequate self-care level. The SCHFI 6.2 [34] will also be used to assess the self-care maintenance at 6, 9, and 12 months from enrollment. Several additional secondary outcomes will be evaluated at a patient level using the measurements described below.

The Heart Failure Somatic Perception Scale is the instrument used to measure physical HF symptoms. It is an 18-item Likert scale that assesses the extension of the discomfort due to symptoms perceived by patients in the week before assessment. The score for each item can range from 0 (*I did not have the symptom*) to 5 (*extreme discomfort*). Once scores are summed, higher values indicate higher symptoms bothersomely.

The Six Item Screener [35] is adopted to identify cognitive impairment or dementia; patients’ assessment using this tool requires a 3-item recall test, and it only takes 1 to 2 minutes to be administered: the subject should be able to repeat the 3 items initially and recall them after 5 minutes. The number of errors recorded in patients’ answers is used as a cutoff point. The KCCQ [37] assesses physical function, symptoms, social function, self-efficacy, and QoL in patients with HF. The KCCQ is a 23-item Likert scale used to measure the health status responsive to changes in clinical condition. The KCCQ scoring regarding health status can range from 0 to 100: 0-24 indicates very poor to poor; 25-49 poor to fair; 50-74: fair to good; and 75-100 good to excellent.

The MS is a 15-item Likert instrument used to measure mutuality from the patient’s or the caregiver’s perspective. Each item can range from 0 to 4. The mean of all item scores constitutes the overall scale score; higher scores indicate better mutuality. The SF-12 [39] is a practical, reliable, and valid tool adopted to measure physical and mental health status. It is a 12-item short-form health survey with eight domains and 1 or 2 items per domain: physical functioning, role-physical, bodily pain, general health, vitality, social functioning, role-emotional, and mental health. The Physical Component Summary (PCS) and Mental Component Summary (MCS) scores are composed of each health domain score. Scores range from 0 to 100; higher scores indicate better physical and mental health functioning. A score of 50 or less on the PCS-12 determines the physical condition; a score of 42 or less on the MCS-12 indicates “clinical depression.”

The PSQI [40] is a self-report questionnaire used to screen patients for the presence of significant sleep disturbance experienced in the month before the assessment. The measure consists of 19 individual items, creating 7 components that are summed to obtain a global PSQI score ranging from 0 to 21; higher scores indicate worse sleep quality. The HADS [41] is a 14-item measure used to detect anxiety and depression in patients. Items are rated on a 4-point scale. The HADS produces 2 scales, 1 for anxiety (HADS–A) and 1 for depression (HADS–D); anxiety and depression are confirmed by scores equal to or greater than 11.

The MoCA is used to detect mild cognitive dysfunction. It assesses different cognitive domains: attention and concentration, executive functions, memory, language, visuoconstructional skills, conceptual thinking, calculations, and orientation. Scores on the MoCA range from 0 to 30; a score of 26 and higher is considered normal. The CCI [43] predicts 10-year survival in patients with multiple comorbidities. The updated index of 12 comorbidities predicts in-hospital mortality; the severity of comorbidity is categorized into 3 grades: mild, moderate, and severe, with CCI scores of 1-2, 3-4, and ≥5, respectively.

The following tools have been used to evaluate the caregivers’ specific secondary outcomes. The CC-SCHFI [44] comprehends 10 items that assess the caregiver’s contribution to maintaining a stable condition and 10 items that assess the caregiver’s ability to monitor and recognize symptoms of HF decompensation, implement adequate treatments, evaluate those treatments, and help patients to take part in each phase of the self-care process. Each measure uses a 4-point Likert response scale with a score from 0 to 100; higher scores indicate a higher contribution to self-care.

The Caregiver Preparedness Scale is an instrument that consists of 8 items that ask caregivers how prepared they feel to handle multiple domains of caregiving. Responses are rated on a 5-point Likert scale with scores ranging from 0 to 4. The scale is scored by calculating the mean of all items. Higher scores indicate higher caregiver preparedness. The Multidimensional Scale of Perceived Social Support is a 12-item scale of perceived appropriateness of the support offered by family, friends, and significant other. The measure uses a 5-point Likert scale. The score can range from 12 to 84, and a higher score indicates greater social support perceived by the caregiver.

### Intervention and Participant Timeline

All patients meeting the inclusion criteria and not any exclusion criteria will be randomized by the researchers responsible for the study after having signed a consent form. Patients will be informed about the single allocation and will not be aware of the randomization list and allocation sequence. The intervention will be administered by nurses trained in HF self-care and MI.

**Figure 1:**
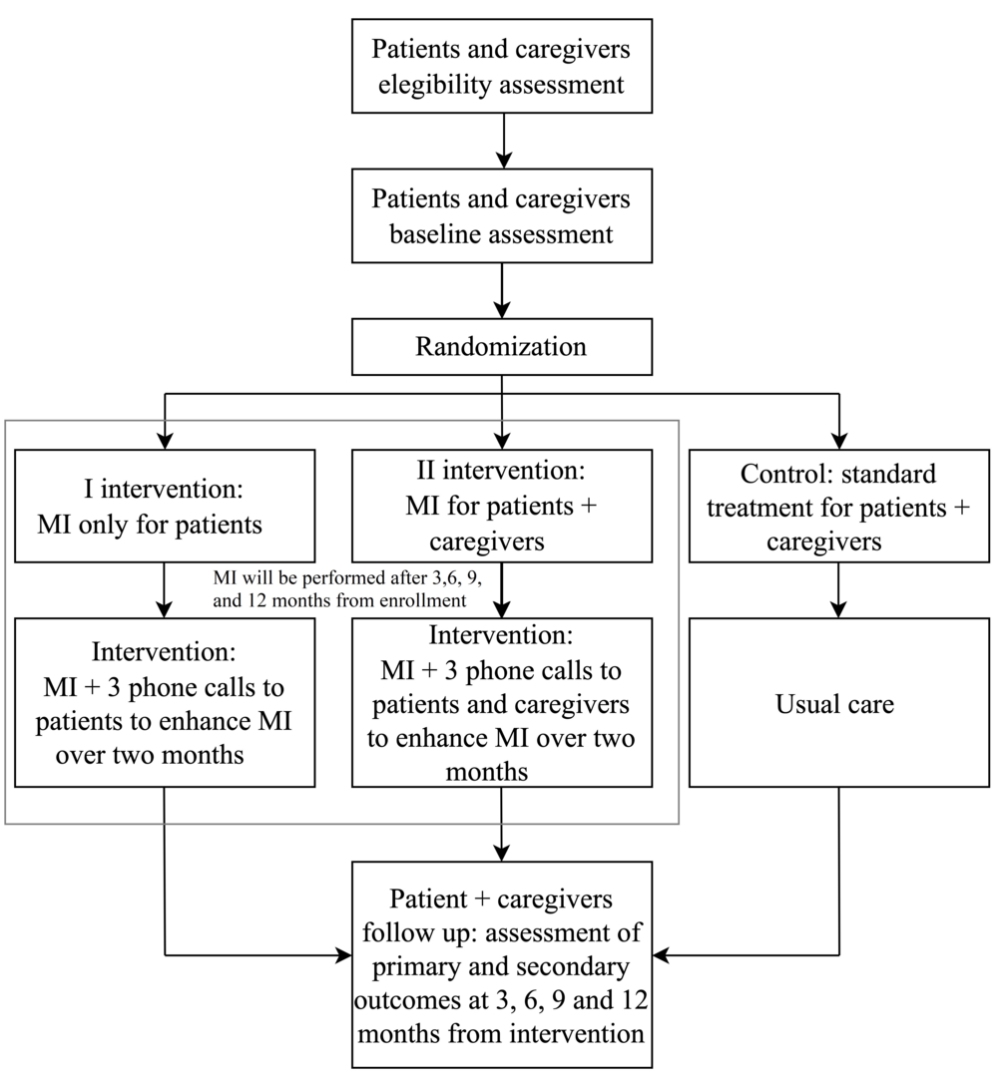
Study flowchart. MI: motivational interviewing.

The study flowchart is shown in Figure 1. In the first interventional arm, the MI intervention will be administered to patients only, and caregivers will receive standard education. MI is used to reinforce areas of inadequate self-care (eg, low physical activity or not having a low-salt diet) and to agree with the patients on the steps to be adopted to modify their behavior. MI focuses on a tailored patient approach to improve patients’ adherence to self-care behaviors. The intervention will last approximately 30 minutes. Thereafter, the nurse who performed the MI will contact the patients over the phone 3 times every 2 weeks from the first MI during the first 2 months after MI to strengthen the intervention. Then, the intervention will be used again after 3, 6, 9, and 12 months from enrollment. After each MI session, both patients and caregivers will be asked to complete the follow-up questionnaires post enrollment as described in the “Instrument” section. In the second interventional arm, MI intervention will be administered both to patients and caregivers. The intervention will be administered to the dyad in 1 session, and patients and their caregivers will receive the reinforcing educational treatment calibrated on the specific lacking areas of patients’ self-care behaviors. After each MI session, both patients and caregivers will be asked to complete the follow-up questionnaires post intervention. In the control group, the dyads will receive only traditional educational treatment and complete the questionnaires after 3, 6, 9, and 12 months of follow-up post intervention. Enrolled dyads will be monitored by the research team responsible for recruitment and follow-up.

### Training for Nurses

Nurses will participate in a training program to acquire the competence to conduct a MI. The education program will be provided to nurses by an expert psychologist.

### Sample Size Calculation

For determining sample size, based on a recent literature review [33], it was hypothesized that both experimental arms would show an improvement in self-care maintenance, and the adequacy of self-care maintenance scores in the experimental arm was shown in approximately 50% (n=250) of patients. Current Italian data indicated that roughly 20% (n=238) of Italian adults with HF reported adequate scores of self-care maintenance (scores higher than 70 are considered adequate) [47,48]. For this reason, the sample size estimation was based on a 2-sample proportions test: the expected proportion in experimental arms was equal to 50% of adequate scores (proportion 1) versus 20% of the adequate score in the control group based on the current descriptive literature (proportion 2, delta=30%). Setting alpha at .05 and power at 0.90, the number per group to identify significant differences was equal to 52. Therefore, as the study has 3 arms, 156 participants are required to test the differences in proportions between groups. Given the likelihood of a 15% rate of dropouts as per previous studies [32], the total sample size was equal to 180 diads (n=60 per arm). A consecutive screening and enrollment will be performed.

### End Points

The end points of this study are designed for the specific aim focused on testing the efficacy of a structured program, including scheduled MI interventions, in improving self-care maintenance. Precisely, the primary end point is the difference between self-care maintenance levels between the 2 groups in the 3-month follow-up. The secondary end points are the differences between self-care maintenance, self-care monitoring, and self-care management at 6, 9, and 12 months of follow-up after enrollment.

### Statistical Analysis

Analyses will be performed within the framework of intention-to-treat analysis. Sociodemographic and clinical characteristics will be summarized and compared between groups in the baseline. For the primary end point, the differences between proportions of adequate self-care maintenance in the 3-month follow-up will be assessed using the chi-square test (or the Fisher exact test if adequate) and performed to compare the first experimental arm (MI delivered only to patients) and control group and between the second experimental arm (MI delivered to the dyad) and control group.

In addition, beyond the comparison between proportions aimed to detect clinically meaningful differences between groups, the standardized 0-100 scores of self-care maintenance will be compared using a 1-way ANOVA with post hoc comparisons adjusting the significance according to Bonferroni. A stepwise logistic regression model based on a dichotomic outcome (adequate self-care vs inadequate self-care scores) will be used to encompass the effects of clinical and sociodemographic covariates in determining the association between the group and the proportion of adequate self-care. The first step of the stepwise approach will include the trial group as an independent variable to determine the odds ratios, highlighting the associations between each group and the dichotomic outcome. The second step will allow authors to include the covariates that show significant linear relationships with the outcome to adjust the associations described in the first step. This model will be evaluated using goodness-of-fit tests, such as the Hosmer-Lemeshow test. The same approach will be used by considering the standardized 0-100 scores of self-care maintenance as the dependent variable and, therefore, in the framework of multiple linear regression. While the logistic regression will provide an association between the adequacy of self-care maintenance and the arm by accounting covariates for adjusting the estimate, the multiple linear regression will highlight the amount of variance explained by belonging to each arm, accounting for the same covariates used in the logistic regression.

The analytical strategy for testing the secondary end points will be the same adopted for the primary end point. In addition, in the framework of time-to-event analysis, the cumulative probability of shifting from inadequate to adequate self-care (ie, the event will be defined as each shift from self-care maintenance scores under 70 to scores equal to or greater than 70) at 6, 9, and 12 months of follow-up after enrollment will be described using Kaplan-Meier curves and inferentially tested using log-rank tests.

The aims focused on testing the effect of the structured program based on MI interventions on secondary outcomes (eg, self-care monitoring, QoL, sleep disturbance) at 3, 6, 9, and 12 months from the enrollment and corroborating the superiority of the caregivers’ participation to the MI interventions over the MI interventions administrated only to individual patients in enhancing self-care behaviors are not used for determining specific end points. Therefore, the analytical plan for assessing those aims will be based on exploratory analyses based on comparing the secondary outcomes scores between the study arms.

In the case of missingness, analyzing the extent of the missingness and identifying underlying mechanisms and patterns will guide imputation methods. If missing at random criteria will be plausible, multiple imputations based on random effect models will be considered for missingness >5% of the outcome assessments at each time point of the trial. Inferential analyses will be based on an alpha of 5% and 2-tailed null hypotheses using Stata 17 (StataCorp).

### Ethical Considerations

The study protocol and template consent forms have been reviewed and approved by the Ethical Committee of San Raffaele Hospital (approval #74/INT). Also, the study protocol has been registered at ClinicalTrials.gov (NCT05595655). Any amendments to the protocol will be submitted to the committee. Patients and caregivers will have to sign the informed consent forms before the randomization. Patients and caregivers may leave the study at any time. The withdrawal from the study will not imply any prejudice, as fully documented and explained in the informed consent. The full protocol will be available freely.

## Results

The data collection was started in May 2017. We completed the data collection with the last follow-up in May 2021. We plan to perform data analysis by December 2022. We plan to publish the study results within March 2023.

### Dissemination

Data collected during the study will be considered the property of the principal investigator and the promoter. Participants’ sensitive data will be processed according to the current privacy legislation. Study results may be published in peer-reviewed journals or submitted to relevant national and international conferences. The data monitoring committee will be constituted of professional monitors identified by the institution and the principal investigator. The monitoring process will encompass all the procedures to guarantee proper transparency and study conduction. No competing interests will be presented by the data monitoring committee.

## Discussion

The general aims of this RCT will be to test the effectiveness of a program of MI interventions in improving self-care behaviors among patients with HF and the caregiver's contribution to self-care and to assess the extent to which caregivers' participation in a program of MI interventions impacts on enhancing self-care behaviors employed by patients. Moreover, this study aims to describe the patient's clinical course and to evaluate the persistence of the results achieved over 1-year time. Several secondary outcomes resulting from questionnaires administered to patients and caregivers will be considered as well, such as sleep quality [40] or HF-specific QoL [7]. Although this study may seem like the Motivational Interviewing to Improve Self-care in Heart Failure Patients (MOTIVATE-HF) study [32], we would like to highlight a difference detectable in the intervention setting administered to patients and caregivers. In fact, in this RCT the interventionist will meet up with the dyad 5 times over 1 year, while in the MOTIVATE-HF study [32] the dyad met up face-to-face with the interventionist only once, and the intervention was bolstered via phone calls. We believe that an intervention including more face-to-face administration would be more powerful in acting on the outcomes of interest.

Although MI is effectively largely used either alone or combined with other treatments and is administered in different settings and ways (ie, patients’ homes; over the telephone; and in hospitals, clinics, or nursing homes), face-to-face interventions seem to be more effective [49]. There is rationale to believe that a more intensive intervention program would even ameliorate and better support patients’ self-care behaviors. Moreover, involving caregivers in this intensive intervention program would make them feel more participatory and aware of the patient’s care process, recognizing their pivotal role in disease management and enhancing their knowledge about HF disease. Besides, dyads with higher shared HF knowledge are more efficient in promoting self-care adherence behaviors [29]. Moreover, both patients and caregivers may perceive proximity with health care professionals, resulting in a better ability to follow the received health professionals’ directions [50]. Self-care is built up on attitudes, behaviors, knowledge, and self-efficacy, and the literature suggests that HF self-care management is more challenging than self-care maintenance and is enhanced by the presence of a caregiver who needs to be properly trained and involved [50,51].

This study protocol presents some limitations that need to be pointed out. First, the monocentric design could affect the generalizability of the results, and the nature of the intervention does not permit a double-blind study design. Moreover, the rather short follow-up (1 year) does not give correct estimates of the lifetime effects of the intervention. Lastly, study participants might be healthier than the average person with HF, as they are usually health conscious with a better lifestyle.

The COVID-19 pandemic still causes mobility restrictions for the population to control infection spread; in particular, people living with a disease, such as patients with HF, are forced to be followed up with remotely by health care professionals to reduce the risk of infection [52,53]. This paradigm shift based on telemedicine and remote monitoring may be fundamental to preventing HF from worsening during circumstances discouraging outpatient visits [52]. However, when circumstances permit, patients’ encounters with health care professionals are recommended to guarantee a periodic proper assessment for early signs of worsening disease [54]. The in-person meetings with patients and caregivers will be exploited to administer MI, respecting all the safety regulations for infection containment.

### Conclusion

MI potentially enhances self-care practices in patients with HF and their caregivers. The conduction of this study may support changes in clinical practice to include MI to enhance the self-care of patients with HF.

## Appendix

Multimedia Appendix 1 SPIRIT (Standard Protocol Items: Recommendations for Interventional Trials) 2013 checklist.

## Abbreviations

CCI: Charlson Comorbidity Index
CC-SCHFI: Caregiver Contribution to Self-care of Heart Failure Index
HADS: Hospital Anxiety and Depression Scale
HF: heart failure
KCCQ: Kansas City Cardiomyopathy Questionnaire
MCS: Mental Component Summary
MI: motivational interviewing
MoCA: Montreal Cognitive Assessment
MOTIVATE-HF: Motivational Interviewing to Improve Self-care in Heart Failure Patients
MS: Mutuality Scale
NYHA: New York Heart Association
PCS: Physical Component Summary
PSQI: Pittsburgh Sleep Quality Index
QoL: quality of life
RCT: randomized controlled trial
SCHFI 6.2: Self-Care of Heart Failure Index 6.2
SF-12: 12-Item Short Form Survey
SPIRIT: Standard Protocol Items: Recommendations for Interventional Trials

## Data Availability

All data relevant to the study are available on reasonable request to the corresponding author.
